# Genes and Pathways Involved in Adult Onset Disorders Featuring Muscle Mitochondrial DNA Instability

**DOI:** 10.3390/ijms160818054

**Published:** 2015-08-05

**Authors:** Naghia Ahmed, Dario Ronchi, Giacomo Pietro Comi

**Affiliations:** Neurology Unit, IRCCS Foundation Ca’ Granda Ospedale Maggiore Policlinico, Dino Ferrari Centre, Department of Pathophysiology and Transplantation, Università degli Studi di Milano, via Francesco Sforza 35, Milan 20122, Italy; E-Mail: naghia.ahmed89@gmail.com

**Keywords:** mtDNA maintenance, multiple mtDNA deletions, adult mitochondrial disorders, progressive external ophthalmoplegia, mitochondrial myopathy

## Abstract

Replication and maintenance of mtDNA entirely relies on a set of proteins encoded by the nuclear genome, which include members of the core replicative machinery, proteins involved in the homeostasis of mitochondrial dNTPs pools or deputed to the control of mitochondrial dynamics and morphology. Mutations in their coding genes have been observed in familial and sporadic forms of pediatric and adult-onset clinical phenotypes featuring mtDNA instability. The list of defects involved in these disorders has recently expanded, including mutations in the exo-/endo-nuclease flap-processing proteins MGME1 and DNA2, supporting the notion that an enzymatic DNA repair system actively takes place in mitochondria. The results obtained in the last few years acknowledge the contribution of next-generation sequencing methods in the identification of new disease loci in small groups of patients and even single probands. Although heterogeneous, these genes can be conveniently classified according to the pathway to which they belong. The definition of the molecular and biochemical features of these pathways might be helpful for fundamental knowledge of these disorders, to accelerate genetic diagnosis of patients and the development of rational therapies. In this review, we discuss the molecular findings disclosed in adult patients with muscle pathology hallmarked by mtDNA instability.

## 1. Introduction

Mitochondrial disorders display heterogeneous clinical presentations in terms of age at onset, progression and symptoms. This variability reflects their complex pathogenesis, which might affect structural proteins and enzymes involved in oxidative metabolism, finally leading to the mitochondrial dysfunction observed in multiple cell types and tissues [1].

Serum abnormalities (*i.e.*, increased lactate levels) or evidence of respiratory chain impairment in affected tissues (*i.e.*, isolated or combined complex deficiency) are often indicative of inadequate mitochondrial respiration, although they are not observed in many primary mitochondrial disorders [2]. Moreover, they lack specificity or should be tested in inaccessible tissues, jeopardizing their efficacy in a clinical setting. Therefore, clinical variability and the unavailability of reliable biomarkers delay the diagnosis in early- and late-onset mitochondrial disorders.

In adult non-syndromic clinical presentations, the involvement of skeletal muscle is mainly exhibited by external ophthalmoplegia, isolated or accompanied by limb weakness [3]. In these patients, histological studies on muscle biopsy usually reveal the presence of fibers not reacting to cytochrome c oxidase staining (COX-negative fibers) or showing signs of compensatory mitochondrial proliferation (ragged-red fibers) [4]. Southern blot analysis of muscle-extracted mitochondrial DNA (mtDNA) can detect reduced mtDNA content and multiple deletions, reflecting mtDNA instability [5]. Although subtle mtDNA deletions can accumulate in the muscle of patients harboring mtDNA point mutations [6], they are largely considered hallmarks of adult disorders due to defects in nuclear genes. Therefore, the disclosure of these alterations is helpful to identify a specific subgroup of mitochondrial disorders (multiple mtDNA deletions syndromes), but the large number of imputable genes hampers the chance of a prompt molecular diagnosis, especially in sporadic cases.

In 2000, missense mutations in *SLC25A4* (encoding for the mitochondrial translocator *ANT1*) have been identified as the first molecular defects resulting in impaired mtDNA maintenance in human [7]. Since then, the hunt for molecular defects underlying multiple mtDNA deletion syndromes has demonstrated to be a very active field of research contributing to improving the diagnostic yield of these conditions. At the same time, these studies expanded our knowledge of the proteins involved in the replication and repair of the mitochondrial genome. Some of these proteins were found to have multiple, sometimes redundant activities, while others seem to have unique functions [8].

In this review, we overview the genes hosting mutations linked with adult-onset phenotypes featuring the accumulation of mtDNA deletions in skeletal muscle. The limited number of patients harboring mutations in the most recently-described genes prevents the definition of clear genotype-phenotype correlations, which are still elusive, even for the most frequent genetic defects. However, the systematic collection of clinical and molecular data of diagnosed patients might help to disclose features shared by subjects harboring mutations in the same gene or pathway.

## 2. Molecular Features in Adult Mitochondrial Disorders Featuring Muscle mtDNA Instability

Disturbances of mtDNA homeostasis result in clinical presentations affecting every stage of life. Infantile and pediatric forms are mostly associated with a strong reduction of mtDNA content in affected tissues; therefore, they are collectively termed mtDNA depletion syndromes [9]. The low residual mtDNA levels cannot sustain a proper respiratory chain assembly: the resulting dysfunction mainly impacts complexes containing mtDNA-encoded subunits and spares complex II, which is entirely encoded by nuclear DNA.

In adults, large-scale deletions of mtDNA spontaneously accumulate in post-mitotic tissues with ageing [6]. The position and extension of such deletions within the mitochondrial genome may be heterogeneous, and in most cases, their accumulation does not result in clinical presentations, although a biochemical defect could be observed by histochemical or enzymatic studies. These slight alterations might also be involved in muscle weakness naturally occurring with ageing. On the opposite side, in primary mitochondrial disorders due to impaired mtDNA maintenance, deletions tend to accumulate in post-mitotic tissues (*i.e.*, muscle) and result in clinical phenotypes, even in relatively young subjects [10]. These alterations are easily detectable on muscle-extracted DNA specimens using Southern blot analysis and standard or quantitative PCR protocols. The co-existence of a quantitative (depletion) and qualitative (deletions) alteration of mtDNA in the same patient is not excluded.

**Figure 1:**
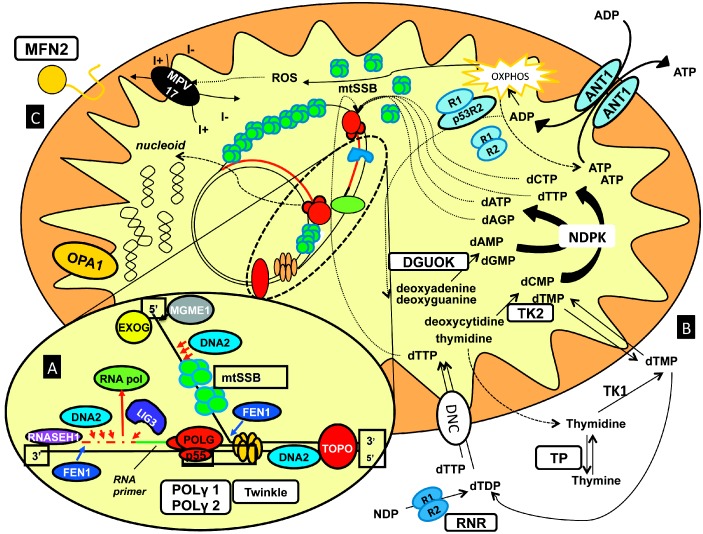
Schematic overview of the proteins and pathways involved in mtDNA maintenance. Zooming in on the mtDNA allows the identification of factors involved in mtDNA replication and repair (**A**) including: POLG, Twinkle, DNA2, MGME1; on the right (**B**) are the proteins assumed to affect the import and metabolism of the mitochondrial dNTP precursors; on the left (**C**) are the supposed localization of key factors ruling mitochondrial dynamics (MFN2, OPA1).

Both pediatric and adult-onset presentations due to mtDNA instability originate from molecular defects in nuclear genes. This is relevant for human health, since such disorders are transmitted according to Mendelian inheritance. Infantile- or juvenile-onset mtDNA depletion syndromes are associated with recessive mutations, resulting in the loss of function of the encoded enzymes. Adult presentations are genetically more heterogeneous, presenting both recessive and dominant types of transmission, these two forms co-existing even for the same gene [11] (Figure 1).

The number of genes involved in this class of disorders has rapidly increased in the last few years, mostly due to the results achieved using next-generation sequencing protocols in familial and sporadic cases. Although the encoded proteins might display multiple catalytic activities and the functional characterization is incomplete for most of them, a (provisional) classification of the genes involved in human mtDNA instability disorders can be attempted (Table 1):

(1) Genes encoding for key proteins of the core mtDNA replication machinery: *POLG* [12], *POLG2* [13], *PEO1* [14];
(2) Genes encoding for proteins involved in mtDNA repair and maintenance: *DNA2* [15], *MGME1* [16];
(3) Genes encoding for proteins preserving the mitochondrial nucleotide pool: *TP* [17], *TK2* [18], *DGUOK* [19], *SLC25A4* [7], *RRM2B* [20], *SUCLA2* [21], *SUCLG1* [22], *ABAT* [23];
(4) Genes encoding for proteins involved in mitochondrial dynamics and remodelling of mitochondrial membranes: *OPA1* [24], *MFN2* [25], *FBXL4* [26,27].

Although mutations of some genes have been so far restricted to infantile (*i.e.*, *FBXL4*) or adult (*i.e.*, *MGME1*) presentations, novel evidence supports the concept of a genetic overlap between these forms, with mutations in the same gene resulting in striking differences in age of onset, tissues affected, progression and outcome. The reason for this heterogeneity is currently unknown. The hypothesis that pediatric presentations could be associated with deleterious mutations, strongly impacting on enzyme activity of the encoded protein, while variants disclosed in adult patients could preserve higher levels of residual activity, is only partially supported by experimental data [28]. The low levels of expression and basal activity of TK2 observed in skeletal muscle might explain the tissue selectivity of TK2 dysfunction, but they are not useful to understand why age of onset and progression are so different between infantile and adult forms [29]. Moreover, the number of pediatric and adult patients sharing the same molecular defects is still low, making genotype-phenotype correlations hazardous or not obvious. It is likely that on-going projects of massive parallel sequencing in novel probands featuring mtDNA instability will further expand this genetic overlap.

The proposed classification does not include all of the genetic defects featuring muscle mtDNA instability. For example, although mutations in *SPG7*, encoding paraplegin, classically result in neurodegenerative diseases, such as hereditary spastic paraplegia [30] and optic neuropathies [31], they have been also described in patients with complex phenotypes showing prominent spastic ataxia with mitochondrial muscle pathology hallmarked by mtDNA deletions [32]. Similarly, mutations in *CHCHD10* have been originally described in familial amyotrophic lateral sclerosis with fronto-temporal dementia [33]. Muscle biopsies of affected subjects revealed respiratory chain dysfunction, COX-negative fibers and a large amount of mtDNA deletions. Following this observation, muscle mitochondrial defects, without signs of mtDNA instability, have been observed in other patients presenting pure motor neuron phenotypes [34,35] or isolated mitochondrial myopathy [36]. The role of CHCHD10 is not completely understood: some evidence indicates that it might take part in mitochondrial dynamics and cristae remodelling [37].

In the following paragraphs, we will focus on genes associated with adult presentations featuring primary accumulation of mtDNA deletions in muscle.

**Table 1:** Genes involved in disorders featuring mitochondrial DNA instability.

Pathway	Gene	Locus	Encoded Protein	Transmission	Onset	mtDNA Defects	Tissues Mainly Affected	Clinical Phenotypes
mtDNA replication	*POLG*	15q25	DNA polymerase gamma, catalytic subunit	AD, AR	Adult	dels	muscle	PEO, MM
				AR	Infantile	depl	liver	MDS, ME, AS
					Adult	depl	cerebellum	MIRAS
	*POLG2*	17q	DNA polymerase gamma, accessory subunit	AD	Adult	dels	muscle	PEO
	*PEO1*	10q24	Twinkle	AD	Adult	dels	muscle	PEO
				AR	Infantile	depl	liver	MDS
					Infantile	depl	brain	IOSCA, ME
mtDNA repair	*DNA2*	10q21.3–q22.1	DNA replication helicase/nuclease 2	AD	Adult	dels	muscle	PEO, MM
	*MGME1*	20p11.23	Mitochondrial genome maintenance exonuclease 1	AR	Adult	dels/depl	muscle	PEO, MM
dNTPs pools maintenance	*SLC25A4*	4q35	Adenine nucleotide translocator	AD	Adult	dels	muscle	PEO
	*TYMP*	22q13	Thymidine phosphorylase	AR	Late childhood Adolescence	dels/depl	muscle	MNGIE
	*TK2*	16q22–q23.1	Thymidine kinase 2	AR	Early childhood	depl	muscle	MDS
				AR	Adult	dels	muscle	PEO, MM
	*DGUOK*	2p13	Deoxyguanosine kinase	AR	Neonatal Infantile	depl	liver/muscle	MDS
				AR	Adult	dels	muscle	PEO
	*RRM2B*	8q23.1	Ribonucleotide reductase M2 B	AR	Infantile	depl	muscle	MDS
				AR	Adult	depl	muscle	MNGIE
				AD	Adult	dels	muscle	PEO
	*SUCLA2*	13q12.2–q13.1	Succinyl-CoA ligase, beta subunit	AR	Early childhood	depl	muscle	MDS
	*SUCLG1*	2p11.2	Succinyl-CoA ligase, alpha subunit	AR	Neonatal Infantile	depl	muscle/liver	MDS
	*ABAT*	16p13.2	4-aminobutyrate aminotransferase	AR	Infantile	depl	brain/muscle	MDS
Mitochondrial dynamics	*OPA1*	3q28–q29	Mitochondrial dynamin-like GTPase	AD	Adult	dels	muscle	OA plus
	*MFN2*	1p36.22	Mitofusin 2	AR	Adult	dels	muscle	OA plus
	*MPV17*	2p23.2	Mpv17 mitochondrial inner membrane protein	AR	Neonatal Infantile	depl	liver	MDS
				AR	Adult	dels	brain	ME
	*FBXL4*	6q16.1	F-box and leucine-rich repeat (LRR) protein	AR	Neonatal Infantile	depl	brain/muscle	ME/

Abbreviations: mitochondrial DNA (mtDNA), autosomal dominant (AD), autosomal recessive (AR), multiple mtDNA deletions (dels), mtDNA depletion (depl), progressive external ophthalmoplegia (PEO), mitochondrial myopathy (MM), mtDNA depletion syndrome (MDS), Alpers’ syndrome (AS), mitochondrial recessive ataxia syndrome (MIRAS), mitochondrial encephalopathy (ME), optic atrophy (OA), mitochondrial neurogastrointestinal encephalopathy (MNGIE), infantile-onset spinocerebellar ataxia (IOSCA).

### 2.1. Genes Encoding for Members of the mtDNA Replication Machinery

The minimum replicative apparatus (replisome) of mtDNA includes the polymerase γ (POLG), the only DNA polymerase active within mitochondria of animal cells, the hexameric helicase Twinkle and the single-strand binding proteins (mtSSBPs). Other enzymatic activities, likely essential for mtDNA replication, include POLRMT, which supplies primers to start replication at the origin of the heavy strand, RNASEH1, which removes primers used during the synthesis of lagging strand, and TOP1MT, a 72-kDa topoisomerase, required to relax negative supercoils [38].

#### 2.1.1. *POLG*

The mammalian mitochondrial polymerase γ is a 250-kDa heterotrimer composed of a 140-kDa catalytic α subunit (encoded by human *POLG*) and two 55-kDa accessory β subunits (encoded by human *POLG2*) [39]. It is synthesized as a precursor containing an amino-terminal leader sequence, which targets the protein towards mitochondria, where the precursor is cleaved. The catalytic subunit contains a carboxy terminal polymerase domain (*pol*) and an amino terminal 3′–5′ exonuclease domain (*exo*) with proofreading activity, separated by a linker region [40]. The intrinsic exonuclease 3′–5′ function greatly improves the replication fidelity, and it is necessary to suppress mtDNA deletions between direct repetitions [41].

Mutations in α subunits result in pediatric- and juvenile-onset presentations such as Alpers–Huttenlocher syndrome (severe infantile-onset encephalopathy with epilepsy associated with hepatic failure), SANDO (sensory ataxic neuropathy, dysarthria and ophthalmoplegia) and MIRAS (mitochondrial recessive ataxia syndrome) [42]. The analysis of muscle mtDNA reveals reduced mtDNA content and multiple deletions. *POLG* mutations also constitute the most frequent cause of familiar (dominant or recessive) and sporadic chronic external ophthalmoplegia [12]. In recessive forms, peripheral neuropathy is frequent and might occur decades before ptosis; the onset is precocious compared to dominant forms [43]. Beside muscle, *POLG* mutations often strike the adult central nervous system with presentations including cognitive impairment, dementia and obsessive disorders [44]. *POLG* defects have been documented in different clinical presentations sharing ataxia [45]. Parkinsonism accompanying progressive external ophthalmoplegia (PEO), ptosis and neuropathy was also found to segregate with *POLG* mutations in a few families [44,46,47]. Therefore, *POLG* defects should be regarded as a secondary genetic cause of Parkinson’s disease, with affected patients presenting earlier onset and variable response to levodopa. Few reports also described premature ovarian failure with Parkinsonism and PEO in women harboring *POLG* variants [48,49].

The A467T mutation is the most common pathogenetic substitution in *POLG*, and it is estimated to occur in 36% of the mutated alleles. Its frequency varies between 0.2% and 1% in the general European asymptomatic population [50]. Almost all of the dominant *POLG* mutations associated with PEO are mapped on the polymerase domain of the enzyme. Mutated enzymes compete against wild-type proteins for binding to the replicative fork [51]. Experiments in *S. cerevisiae* displayed a similar behavior between human *POLG* mutations and the corresponding substitutions of the orthologue *mip1*: the severity of yeast phenotypes correlated with the age of onset of human presentations [52].

#### 2.1.2. *POLG2*

The p55 β (accessory) subunits of polymerase γ are encoded by *POLG2*. They constitute a homodimer that binds asymmetrically to the catalytic portion of the holoenzyme (encoded by *POLG*). The proximal subunit strengthens DNA binding, while the distal subunit facilitates the nucleotide incorporation. The disruption of the interaction between the p55 accessory subunit and the p140 catalytic part might promote the stalling of the replication fork and produce mtDNA deletions. Furthermore, POLG2 has a role in nucleoids’ structure [38,53].

Few pathogenetic mutations have been so far identified in *POLG2* (G451E, G416A, c.1207_1208ins24, R369G), the patients presenting autosomal dominant ptosis and PEO with onset in the third to fourth decade, mild proximal muscle weakness with exercise intolerance and other neurologic or systemic symptoms, similarly to *POLG*-mutated PEO patients [13,54,55].

#### 2.1.3. *PEO1*

Also known as *C10orf2*, *PEO1* encodes for a hexameric helicase of the RecA-type superfamily, named Twinkle. It has structural similarities with the gp4 protein of T7 phage primase/helicase. The linker region, important for subunit interactions and the establishment of the functional hexamer, is known to be a mutational hotspot for dominant PEO [14]. Apart from 5′–3′ helicase activity, Twinkle is essential for the maintenance and the regulation of mtDNA copy number [56].

Mutations that suppress Twinkle helicase activity also compromise mtDNA replication and transcription, since the progression of the replicative fork is hampered, causing the accumulation of replication intermediates [57]. The presence of a cluster of mutations in small highly-conserved regions supports the proposed negative-dominant behavior of Twinkle mutations on mtDNA maintenance and transcription [43]. As a consequence, *PEO1* mutations are inherited according to an autosomal-dominant fashion. Heterozygous mutations might also arise *de novo* in sporadic patients [58]. Fratter and colleagues studied a cohort of 33 mutated patients (26 probands) presenting either missense mutations or in-frame duplications [59].

*PEO1*-mutated patients show variable onset ranging from the second to the eighth decade. Clinical features might include ptosis, ophthalmoparesis, proximal hyposthenia, ataxia, peripheral neuropathy, bulbar signs, cardiomyopathy, endocrine disorders, cataract and depression or avoiding personality tracts. A syndromic presentation, including sensory neuropathy with ataxia, dysarthria and ophthalmoparesis (SANDO), can also occur [60]. As for *POLG* mutations, *PEO1* defects have been found to segregate with Parkinsonism and additional syndromic features in dominant PEO families [61,62]. Recessive mutations in *PEO1* are less frequent and result in severe pediatric presentations, including IOSCA (infantile-onset spinocerebellar ataxia), mostly occurring in Finnish patients [63], and a hepatocerebral form of mtDNA depletion syndrome [64].

### 2.2. Genes Encoding for Factors Involved in mtDNA Repair

The accumulation of DNA damage is thought to play a critical role in the aging process, as well as mitochondrial disorders [65]. It was thought for many years that mitochondria lacked an enzymatic DNA repair system comparable to that in the nuclear compartment. However, it is now well established that DNA repair actively takes place in mitochondria, preserving genomic integrity through oxidative DNA damage processing, base excision repair (BER) pathways and further, still uncharacterized, mechanisms [66]. It is likely that the enzymatic activities required for repairing specific mtDNA damage might be also helpful in the processing of replication intermediates, narrowing the functional border between these two groups of proteins involved in mtDNA maintenance. The BER pathway engages the recognition and removal of deaminated, oxidized and alkylated DNA bases. Following the recognition of the damaged base, a group of endo/exonucleases catalyzes the consecutive steps of this orchestrated process. The nuclear BER has been largely explored, as well as the consequences of its dysfunction for human health [67]. Mammalian mitochondria also possess BER activities, promoted by a set of enzymes presenting double localization (nuclear and mitochondrial) or exclusively targeted to the mitochondrial compartment [68]. Interestingly, clinical presentations have been recently associated with altered mtDNA maintenance due to defective mitochondrial endo-/exo-nuclease activities.

#### 2.2.1. *DNA2*

This gene has been recently considered as a candidate for the molecular screening of familiar or sporadic cases with PEO and/or myopathy accompanied by muscle accumulation of mtDNA deletions [15]. Human *DNA2* encodes for a member of the nuclease/helicase family. It was found in mammalian mitochondria, where it participates in the removal of RNA primers during mtDNA replication [69], but exclusive mitochondrial localization is debated [70]. The encoded protein interacts with polymerase γ and stimulates its catalytic activity [69]. As a member of the nuclease/helicase family, human DNA2 contains conserved nuclease, ATPase and helicase domains.

Experimental studies have demonstrated a role for DNA2 in the mitochondrial long patch base excision repair pathway primed by mtDNA defects due to oxidation, alkylation and hydrolysis. In particular, the nuclease activity is involved in the processing of flap intermediates occurring during the removal of damaged bases in BER. Inside mitochondria, this task is accomplished with the partnership of the flap structure-specific endonuclease 1 (FEN1). DNA2 seems also involved in the processing of Okazaki fragments, as demonstrated in yeast [71]. The function of DNA2 as a DNA helicase is less demonstrated. In yeast, helicase activity facilitates the formation of flap intermediates *in vitro* [72], but it was found to be dispensable *in vivo* [73]. Similarly, the mutations identified in patients and localized within nuclease and ATPase domains also impair helicase activity, as shown by *in vitro* studies [15]. Therefore, the helicase activity of DNA2 is also likely involved in mtDNA maintenance. At present, Twinkle remains the only established replicative helicase inside mitochondria. However, DNA2 might partially support Twinkle, since it co-localizes with Twinkle in nucleoids and its mitochondrial recruitment is induced by *PEO1* mutations [74].

A *DNA2* homozygous out of frame truncating mutation has been identified as a genetic cause of Seckel syndrome [75], a disorder characterized by *in utero* and postnatal growth retardation, intellectual disability, microcephaly, facial dysmorphisms and, rarely, cardiac malformations. Enhanced senescence with a marked increase of damaged nuclear DNA has been observed in patients’ fibroblasts, supporting the hypothesis that DNA2 repair activity is affected in this disorder [75]. While these findings acknowledge a nuclear role for human DNA2, the striking differences between pediatric and adult presentations are less clear. The heterozygous mutations disclosed in adult patients impair DNA2 activity less severely with respect to the homozygous truncating mutation linked with Seckel syndrome. Alternative nuclear and mitochondrial factors, replacing defective DNA2 and their differential expression among tissues, might also modulate the phenotype.

#### 2.2.2. *MGME1*

This gene, previously known as *C20orf72*, encodes a RecB-type exonuclease belonging to the PD-(D/E)XK nuclease superfamily [76]. Cell fractioning experiments showed that the encoded polypeptide is targeted to mitochondria, where it is involved in mtDNA maintenance, promoting the turnover of the replication intermediates 7S. MGME1 cuts DNA, but not RNA or DNA-RNA hybrids; it seems to require free 5′-ends to exert its function (it does not work on circular DNA), and it has a greater affinity for single DNA than double-strand DNA *in vitro*. As in the case of DNA2, MGME1 also interacts with pol γ, therefore contributing a further 5′–3′ exonuclease activity to the mitochondrial machinery assembled at the replication fork [77].

Recessive mutations within the *MGME1* coding sequence have been observed in multiple patients from two families and a sporadic patient. Clinical presentation includes external ophthalmoplegia, muscle weakness and a progressive respiratory impairment, requiring assisted ventilation [16].

### 2.3. Genes Encoding for Proteins Maintaining the Mitochondrial dNTP Pool

As for the nuclear genome, mtDNA also require a balanced pool of dNTPs for an effective replication (and repair). In replicative tissues, cytosolic dNTPs, used in nuclear DNA synthesis, can be also imported into mitochondria through dedicated transporters, sustaining mtDNA maintenance. In non-replicative cells, the cytosolic *de novo* synthesis of dNTPs is downregulated, and the mtDNA replication, which persists in post-mitotic cells, relies on a set of intra-mitochondrial reactions to preserve the proportions of deoxyribonucleotides (dNTPs salvage pathway) [78]. Recessive mutations in key enzymes of this pathway might impair the supply of substrates for the mitochondrial DNA polymerase, resulting in defective mtDNA synthesis or increased replicative errors and genomic instability. In quiescent cells, *de novo* dNTP synthesis still partially supports the replication of mtDNA and the repair of the nuclear genome. Therefore, other proteins involved in nucleoside transport and metabolism might be important for mtDNA maintenance, as observed for the cytosolic ribonucleotide reductase (see below) [79]. Besides the obvious role in the supply of the “building blocks” for DNA synthesis, the deregulation of nucleotide levels might also have more complex consequences, overall conditioning the mitochondrial mass in the cell [80].

#### 2.3.1. *TYMP*

Thymidine phosphorylase (TP, encoded by *TYMP*, also known as *ECGF1*) acts as a homodimer catalyzing the phosphorylation of thymidine phosphate to thymidine and 2-deoxy-d-ribose 2-phosphate. The direct reaction is important for nucleosides catabolism, while the reverse reaction participates in the pyrimidine salvage pathway.

Recessive mutations of this gene have been identified as the cause of mitochondrial neurogastrointestinal encephalomyopathy (MNGIE) [17]. These loss-of-function defects result in the reduction (or substantial absence) of thymidine phosphorylase activity and the toxic accumulation of nucleotides in plasma [17]. The imbalance of the dNTP pool increases the rate of mtDNA point mutations, and both mtDNA depletion and deletions are observed at Southern blot analysis of muscle mtDNA. Clinical manifestations depend on residual TP activity. Since *TP* is a homodimer enzyme, the presence of a mutant allele induces 25% wild-type enzyme synthesis: asymptomatic heterozygotes have 25%–35% of enzymatic residual activity, and this threshold represents a target for therapeutic strategies, like liver transplantation, that results in being be six-fold more efficient as a TP source than bone marrow [81]. Neuroradiological studies in *TYMP*-mutated patients often show leukoencephalopathy [82]. Brain MRI has been suggested as a tool for differential diagnosis of *POLG* involvement in recessive PEO presentations [43].

#### 2.3.2. *TK2*

This gene encodes for the thymidine phosphorylase type 2, the enzyme that catalyze the rate-limiting step of deoxypirimidine phosphorylation within the mitochondrial dNTPs salvage pathway. The gene is upregulated in non-replicating cells, where the encoded product safeguards pool availability.

TK2 deficiency has been classically associated with the myopathic form of mtDNA depletion syndrome, featuring an infantile-onset severe phenotype with motor regression and early death due to diaphragmatic paralysis [18]. Recessive mutations have been also reported in presentations resembling spinal muscular atrophy [83]. Mutations in the same gene have been more recently observed in adult phenotypes with ptosis, external ophthalmoplegia, slowly-progressive proximal muscle weakness, muscular atrophy and dysarthria [84,85]. In adult patients with PEO due to *TK2* mutations, multiple deletions, but not depletion, have been found in muscle. The low basal TK2 activity in muscles could explain why most of the clinical features related to TK2 dysfunction affect this tissue [29]. The reason for the different presentations in pediatric and adult subjects is more elusive: indeed, biochemical studies performed in fibroblasts from adult patients with mild myopathy revealed very low TK2 activity levels, not dissimilar to those observed in pediatric cases.

#### 2.3.3. *DGUOK*

*DGUOK* encodes for the mitochondrial deoxyguanosine kinase that catalyzes the first reaction in the purine salvage pathway inside mitochondrial matrix. Recessive mutations have been described in pediatric cases showing severe encephalohepatopathy with severe reduction of liver mtDNA content and premature death, unless the patients undergo a liver transplant [19]. Recently, the massive parallel sequencing of genes encoding for established or predicted mitochondrial proteins (MitoExome) has revealed compound heterozygous mutations even in adult sporadic and familial forms of mitochondrial myopathy [28]. Muscle analysis in mutated patients disclosed mtDNA deletions with variable mtDNA content and significant deficiency of DGUOK protein levels and activity. The main clinical features of adult forms include: muscle weakness, PEO, hearing loss and bulbar signs. One of the probands reported had previously undergone liver transplant. Notably, two siblings harboring *DGUOK* mutations displayed an atypical phenotype characterized by slowly progressive, predominantly distal, upper and lower limb muscle weakness, mild dysphonia and dysphagia, similarly to SMA-like presentations due to TK2 deficiency. These findings clearly demonstrate that the effects of *DGUOK* and *TK2* mutations are not limited to those tissues that are primarily affected in terms of severity and age of involvement.

#### 2.3.4. *SLC25A4*

This gene encodes for the ANT1 member of the ADP/ATP translocator family, localized in the inner mitochondrial membrane. ANT1 predominates in post-mitotic tissues, including skeletal muscle, heart and brain. It acts as a homodimeric gate-channel operating the ADP/ATP exchange between mitochondria and cytoplasm. Since ANT1 senses the adenosine concentration in the two compartments, it is a part of the signaling pathway coupling cellular energy consumption and mitochondrial respiratory chain activity [86]. Moreover, ANT1 is a structural element of the mitochondrial permeability transition pores (MPTP) and has a role in the intrinsic apoptotic pathway [87].

Heterozygous *SLC25A4* mutations have been reported in patients with PEO and ptosis with or without generalized muscle weakness [7]. Cardiac involvement, including cardiomyopathy, disarrangement of myofibers, inflammation linked to heart disease and ischemic attacks [88], has been repeatedly described. Neurosensory hypoacusia, thyroid gout and dementia without affective aspects have also been reported [43]. Mutations in the same gene also result in Sengers syndrome, a multisystemic disorder featuring congenital cataract, hypertrophic cardiomyopathy, mitochondrial myopathy and lactic acidosis. Two forms are known for Sengers syndrome: a neonatal form with poor prognosis and a late-onset presentation with lifespan observed until the third decade [89].

#### 2.3.5. *RRM2B*

This gene maps on chromosome 8q22.1–q23.3 and encodes the minor subunit (p53R2) of ribonucleotide reductase (RNR), the tetrameric enzyme catalyzing *de novo* dNTPs synthesis from the reduction of the corresponding ribonucleosides diphosphate. *RRM2B* expression is tightly regulated by the oncosuppressor p53. In post-mitotic cells, ribonucleotide reductase contributes to mitochondrial dNTP supply [90], in parallel with the salvage pathway.

Recessive mutations within the *RRM2B* coding sequence have been associated with the infantile encephalomyopathic form of mtDNA depletion syndrome [20]. In 2009, linkage analysis was used to drive the discovery of a heterozygous non-sense *RRM2B* mutation in affected members of a big North American family of European origin presenting adult-onset dominant PEO with muscle accumulation of mtDNA deletions. The same mutation in a second family was associated with additional clinical features, including: ataxic gait, hypoacusia, reduction of tendon reflexes and psychiatric disorders [91]. Kearns–Sayre syndrome (KSS) and MNGIE are rarer manifestations. *RRM2B* mutations were observed as the third most common molecular cause of PEO in a large cohort of British adult patients, following *POLG* and *PEO1* [92]. The mutated or truncated protein p53R2 might compete against wild-type protein for the binding of heterodimeric RNR, exerting a dominant negative effect [91], a pathogenic mechanism compatible with defects disclosed in both pediatric and adult subjects.

### 2.4. Genes Encoding for Protein Involved in Mitochondrial Dynamics and Remodeling

It is now well established that intracellular mitochondria are organized in dynamic networks and physiologically undergo remodelling cycling between fission and fusion events [93]. This behavior is crucial for the turnover of aged mitochondria and their intracellular transport, but also compensates the defects accumulating in the organelle as bypass products of oxidative metabolism [94]. Together with mitochondrial protein quality control system, mitochondrial dynamics have emerged as an important field of investigation to address the pathogenesis of human neurodegeneration. Indeed, increased mitochondrial fragmentation due to the reduced ratio between fusion (*i.e.*, MFN2 and OPA1) and fission (*i.e.*, DLP1/DRP1 and FIS1) proteins has been observed in tissues and cell cultures obtained from patients with major neurodegenerative disorders, including Alzheimer’s and Huntington’s disease. Furthermore, Parkinson’s disease has been associated with mutations in PARK2 and PINK1 proteins, which orchestrate mitochondrial protein quality control and the turnover of aged mitochondria by ubiquitination and degradation of MFN2 [95].

#### 2.4.1. *OPA1*

The product encoded by this gene is a regulatory mitochondrial fusion protein localized in the inner membrane, showing GTPase activity [96]. OPA1 is a main regulator of cristae morphology, but also plays a key role in response to cellular cytotoxic insult generated by hyperactivation of *N*-methyl-d-aspartate (NMDA) receptors for an altered homeostasis of mitochondrial calcium. Indeed, the increased agonism of NMDA receptors induces mitochondrial fragmentation and ultrastructural defects of the inner mitochondrial membrane [97]. Conversely, *OPA1* overexpression is protective against cytotoxic glutamate response, with an increased survival of neurons and preserved mitochondrial morphology [98].

Mutations in *OPA1* are the most common cause of isolated optic atrophy with dominant inheritance (ADOA) [99,100], as well as “plus” phenotypes where optic atrophy is accompanied by syndromic features, such as neurosensory deafness and ophthalmoplegia. Muscle from *OPA1* plus patients showed signs of mitochondrial sufferance, including COX-negative fibers and accumulation of mtDNA deletions [24,101]. OPA1 defects not only impact retinal gangliar cells leading to the progressive degeneration of optic nerve, but also nervous and muscle tissues. Some studies have documented a compensatory increase of mtDNA copies in affected muscle fibers, independently of the severity of the disease [102]. The percentage of COX-negative fibers seems to be four-fold superior in the DOA plus form than in pure presentations.

Recently, *OPA1* sequencing in probands from two Italian families affected by dominant chronic PEO complicated by Parkinsonism and dementia revealed two heterozygous missense mutations affecting highly conserved amino acid positions in the GTPase domain [103]. Multiple mtDNA deletions were detected in available muscle biopsies.

#### 2.4.2. *MFN2*

This gene encodes an outer membrane protein with GTPase activity involved in mitochondrial fusion. Molecular defects affecting *MFN2* are a major cause of the Charcot–Marie–Tooth axonal neuropathy type 2A, an autosomal dominant disease characterized by a pronounced impairment of motor and sensory neurons [104].

Mutations in *MFN2* have been recently disclosed in familial multisystemic disorder with optic atrophy beginning in early childhood, associated with axonal neuropathy and mitochondrial myopathy in adult life, a presentation resembling *OPA1* plus phenotype. Mitochondrial DNA deletions were found in muscle while patients’ fibroblasts disclosed defective repair of stress-induced mtDNA damage, leading to respiratory chain impairment [25]. Notably, a detrimental effect on mtDNA replication has been also recently documented in fibroblasts from CMT2A patients, which displayed mtDNA depletion and a minimal amount of deleted molecules [105], suggesting a common pathogenetic mechanism resulting from *MFN2* dysfunction, irrespective of the clinical phenotype.

#### 2.4.3. *MPV17*

This gene encodes for an inner mitochondrial membrane protein whose function has remained obscure for several years. The encoded product is a member of the family of integral membrane proteins comprising PXMP2, MPV17, MP-L and FKSG24 (MPV17L2) in mammals. Studies on vertebrates suggested a role in nucleotide trafficking between mitochondria and cytoplasm [106]. Recently, MPV17 was identified as a non-selective ion channel, responsible for modulation of mitochondrial membrane potential, which is influenced by several conditions in the organelle, such as redox state and protein phosphorylation [107].

Mutations in *MPV17* have been firstly described in a severe juvenile-onset hepatoencephalopathy with major reduction of liver mtDNA content [108]. Coming to adult presentations, recessive *MPV17* mutations have been described in three patients presenting adult-onset multi-systemic disorders with neuropathy and leukoencephalopathy and featuring multiple mtDNA deletions in muscle [109,110,111].

## 3. Genotype-Phenotype Correlations

**Figure 2:**
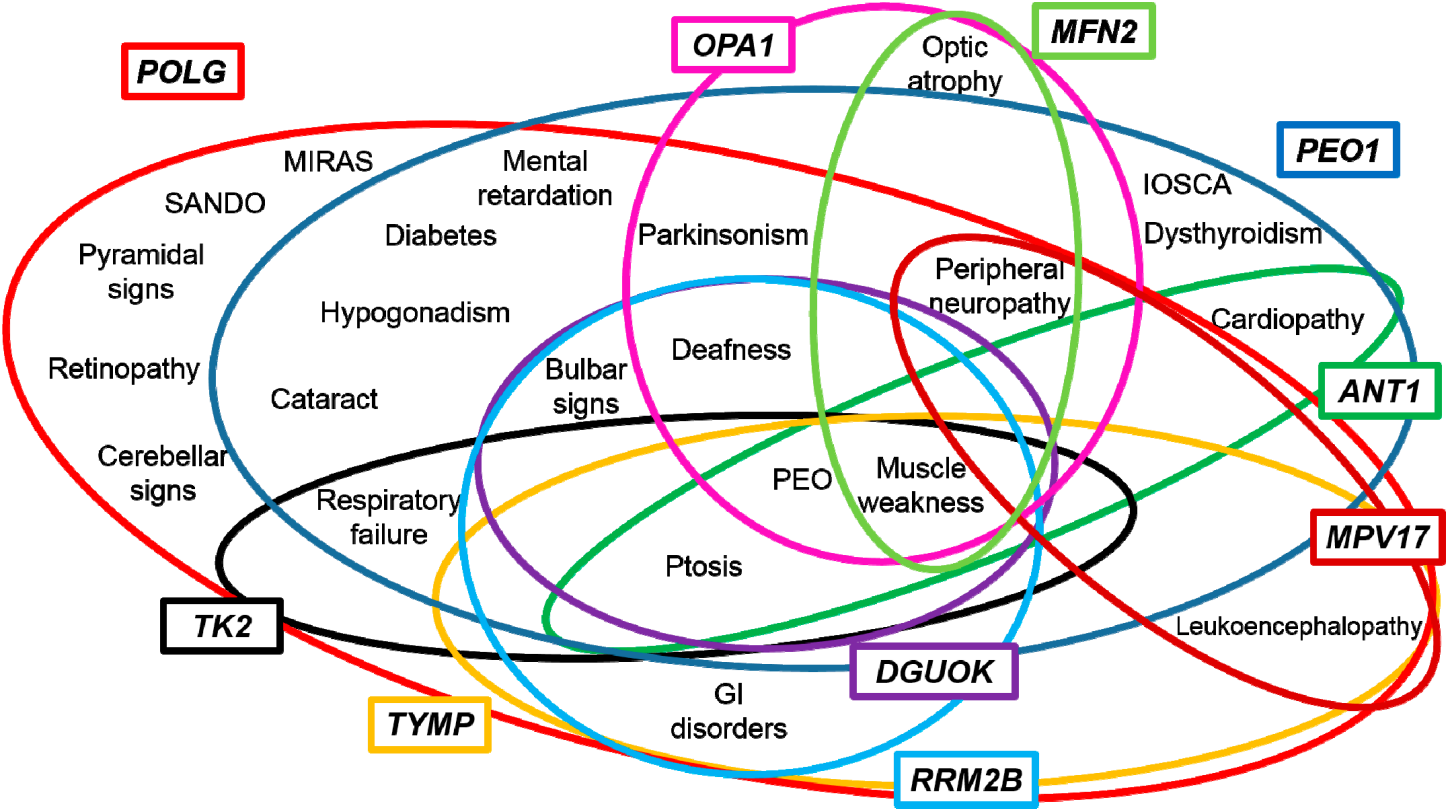
Representation of clinical phenotypes related to nuclear genes involved in mtDNA maintenance. Abbreviations: progressive external ophthalmoplegia (PEO), mitochondrial recessive ataxia syndrome (MIRAS), infantile-onset spinocerebellar ataxia (IOSCA), sensory ataxic neuropathy, dysarthria, and ophthalmoparesis (SANDO).

Mitochondrial disorders display a spectacular clinical heterogeneity. Presentations featuring muscle mtDNA instability, which constitute a tiny subgroup of the spectrum of mitochondrial disorders, do not elude this general consideration. Indeed, the overlapping of clinical features is frequent with heterogeneous genetic defects resulting in undistinguishable phenotypes (Figure 2).

Previous studies reporting a cohort of patients accumulating multiple mtDNA deletions in muscle have attempted genotype-phenotype correlations, with modest results, even considering only forms due to mutations in the same gene [43]. The elucidation of such correlations might be useful to speed molecular diagnosis. The number of candidate genes for molecular tests overcomes the resources of standard laboratories, even if the application of next-generation sequencing in a clinical setting is expected to improve the diagnostic yield, reducing the cost of genetic testing [112]. Despite these issues, some remarks emerge from previous studies. Patients harboring *POLG* mutations display severer clinical features and age-related penetrance, while *ANT1* mutations are associated with milder phenotypes. *PEO1* patients show clinical presentations with intermediate severity of symptoms [43]. Bulbar weakness, deafness and gastrointestinal symptoms are observed in *TYMP* [113] and *RRM2B* patients [92], favoring the analysis of these genes before more frequent defects, such as *POLG* and *PEO1*. Cardiac involvement, reflected by conduction defects and left hypertrophy, is more representative of ANT1 involvement rather than *RRM2B* [92].

Peripheral neuropathy, even if not exclusive, is often associated with *POLG* mutations; however, it also occurs in *PEO1* cases [60]. Recently, a diagnostic flow-chart has been suggested for *POLG*-related diseases in pauci-symptomatic patients affected by peripheral neuropathy and suspected mitochondrial impairment [114]. Peripheral neuropathy has been also frequently observed in ADOA plus patients harboring *MFN2* and *OPA1* mutations [115].

## 4. Towards a Candidate-Pathway Approach

Despite the relevant achievements gained approaching mitochondrial patients with panel or exome sequencing technologies [116,117], the proportion of subjects without a molecular diagnosis is still high. The number of phenotypes reflecting muscle mtDNA instability has increased, while the borders between clinical presentations are getting narrow. As for other Mendelian disorders, the application of next-generation sequencing techniques in a research setting led to the discovery of novel genetic defects [112]. Overall, in only a few years, next-generation sequencing protocols disclosed more genetic defects than any other strategy (linkage studies, gene-candidate approach) in the last two decades. Nevertheless, the number of established diagnosis only slightly increased with a global improvement in the diagnostic yield of around 10%.

In the institutions where panel sequencing is routinely used for diagnostic purposes, the potential reduction of the time-to-diagnosis is challenged by the increased number of variants detected and the consequent efforts required to obtain a reliable genetic and biochemical validation. Taking into account these considerations, a detailed clinical assessment and a more accurate (or extended) genotyping performance might be important, but not decisive advancements. Conversely, the identification of clinical and molecular features shared by patients presenting defects in the same functional pathway, accompanied by quantitative measurements of deleted mtDNA molecules in muscle samples, could be used to drive the selection of genes or pathways to be addressed in molecular testing, improving the diagnosis of single patients in a short timeframe.
